# Severity of *Plasmodium falciparum* and Non-*falciparum* Malaria in Travelers and Migrants: A Nationwide Observational Study Over 2 Decades in Sweden

**DOI:** 10.1093/infdis/jiz292

**Published:** 2019-06-06

**Authors:** Andreas Wångdahl, Katja Wyss, Dashti Saduddin, Matteo Bottai, Elsie Ydring, Tomas Vikerfors, Anna Färnert

**Affiliations:** 1 Division of Infectious Diseases, Department of Medicine Solna, Stockholm; 2 Unit of Biostatistics, Institute for Environmental Medicine, Karolinska Institutet, Stockholm; 3 Department of Infectious Diseases, Västerås Hospital, Västerås; 4 Department of Infectious Diseases, Karolinska University Hospital, Stockholm; 5 Public Health Agency of Sweden, Solna, Sweden

**Keywords:** imported malaria, severe malaria, migrants, Plasmodium, *falciparum*, non-*falciparum*

## Abstract

**Background:**

The aim was to assess factors affecting disease severity in imported *P. falciparum* and non-*falciparum* malaria.

**Methods:**

We reviewed medical records from 2793/3260 (85.7%) of all episodes notified in Sweden between 1995 and 2015 and performed multivariable logistic regression.

**Results:**

Severe malaria according to WHO 2015 criteria was found in *P. falciparum* (9.4%), *P. vivax* (7.7%), *P. ovale* (5.3%), *P. malariae* (3.3%), and mixed *P. falciparum* episodes (21.1%). Factors associated with severe *P. falciparum* malaria were age <5 years and >40 years, origin in nonendemic country, pregnancy, HIV, region of diagnosis, and health care delay. Moreover, oral treatment of *P. falciparum* episodes with parasitemia ≥2% without severe signs at presentation was associated with progress to severe malaria with selected criteria. In non-*falciparum*, age >60 years, health care delay and endemic origin were identified as risk factors for severe disease. Among patients originating in endemic countries, a higher risk for severe malaria, both *P. falciparum* and non-*falciparum*, was observed among newly arrived migrants.

**Conclusions:**

Severe malaria was observed in *P. falciparum* and non-*falciparum* episodes. Current WHO criteria for severe malaria may need optimization to better guide the management of malaria of different species in travelers and migrants in nonendemic areas.

## Teaser

We investigated risk factors for severe malaria in approximately 2700 cases of imported malaria. We identified variables associated with severity of *P. falciparum* and non-*falciparum* infections that need to be considered in the management of malaria in travelers and migrants.

Severe malaria involves dysfunction in 1 or several vital organs, and prompt diagnosis and effective treatment are crucial for the outcome [1]. *Plasmodium falciparum* is the leading cause of severe malaria [2], but severe disease can also be caused by the other species [3–5]. In highly endemic areas, severe malaria affects mainly children before protective immunity is gradually acquired after repeated infections [6]. In nonendemic areas, risk factors for severe *P. falciparum* malaria include nonendemic origin, young and older age, pregnancy, comorbidities such as human immunodeficiency virus (HIV) infection, diabetes mellitus, and obesity [7–12]. In addition, delayed diagnosis and health care presentation in a region where malaria is rarely diagnosed increase the risk of mortality due to malaria [8, 13]. Risk factors for severe non-*falciparum* malaria are not as well described.

The aim of this study was to describe clinical aspects and outcome of imported malaria and to assess factors that affect disease severity in different parasite species using nationwide data in Sweden over 2 decades.

## METHODS

### Study Population

Malaria is a notifiable disease in Sweden, according to the Communicable Diseases Act. All malaria episodes reported between 1 January 1995 and 31 December 2015 in the National Surveillance Database at the Public Health Agency of Sweden, were linked to hospital data through the unique national identity number carried by all residents in Sweden, or the temporary numbers given to visitors or newly arrived migrants upon presentation to hospital. All 28 reporting hospitals contributed with medical records. Additional unreported episodes were identified through microbiology departments and inquiries in the electronic medical records systems of the respective hospitals. Individuals with additional episodes related to new travel or different *Plasmodium* species were regarded as new episodes while relapses and recrudescences were not included in the analysis. All included episodes were microscopy confirmed, generally at the regional departments of microbiology but also by the attending infectious disease specialist.

### Data Collection

Demographic, epidemiological, and clinical data were collected from medical records, including travel details and use of chemoprophylaxis, comorbidities, pregnancy, clinical presentation, health care parameters regarding length of hospitalization, treatments, intensive care unit (ICU), delay to diagnosis, outcome, as well as blood chemistry and microbiology analyses.

### Definitions

Severe malaria was defined according to the World Health Organization (WHO) criteria from 2015 (Supplementary Table 1) [1], with exception of circulatory shock which was exclusively defined as systolic blood pressure <80 mmHg in adults and <70 mmHg in children under 12 years (compensated circulatory shock could not be assessed in the retrospective data). The WHO 2015 definition was extended to include *P. ovale* and *P. malariae* according to criteria defined for *P. vivax.* Severity was also assessed using criteria prognostic of unfavorable outcome in severe malaria, based on selected criteria including impaired consciousness, acidosis, renal impairment and shock, associated with mortality in Bruneel et al and multiple convulsions, pulmonary edema, and significant bleeding in WHO 2000 [14, 15]. Health care delay was defined as number of days from first health care contact until malaria diagnosis.

### Statistical Analysis

The analyses were performed with Stata version 14.2 (StataCorp, College Station, TX). In the descriptive analyses, the categorical variables were summarized by proportions, and the numerical variables were summarized by medians and interquartile ranges (IQR). In comparative analyses, categorical variables were analyzed using the Pearson *Χ*^2^ test, or the Fisher exact test when the latter was computationally possible. The Mann-Whitney U test was used for comparing the distribution of numeric variables between 2 groups and the Kruskal-Wallis test among multiple groups. Univariable logistic regression was used to test potential risk factors for severe malaria, and variables with a *P* value less than .2 were included in the multivariable logistic regression model. The variables with a *P* values less than .05 were kept in the multivariable model. The Wald test was used to test the regression coefficients. As individual patients could appear more than once in the dataset, the standard errors were estimated with the cluster-robust sandwich estimator. *P* values less than .05 were considered statistically significant.

### Ethical Considerations

The study was approved by the Ethical Review Board in Stockholm, Sweden (2009/1328-31/5, 2010/1080-32, and 2012/1155-32).

## RESULTS

**Figure 1.**
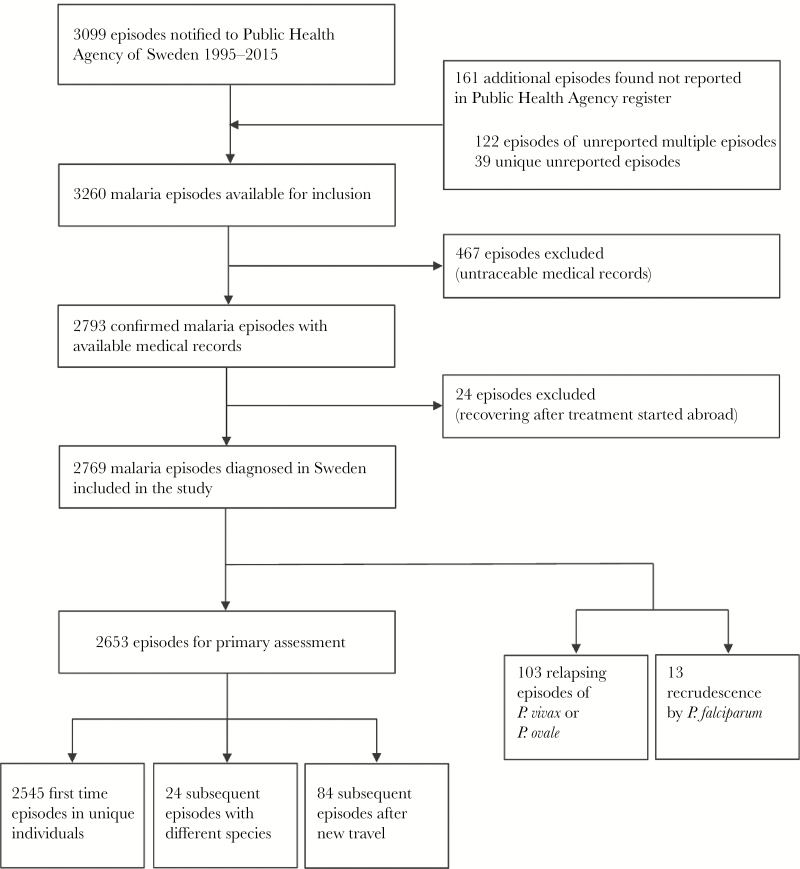
Chart of study population.

During 1995 to 2015, 3099 malaria cases were reported to the Public Health Agency of Sweden; additionally, 161 unreported episodes were identified through hospital data. Medical records were available for 2793 (85.7%) episodes, with missing records from all regions. After exclusion of relapse and recrudescence episodes (n = 116) and reported episodes without microbiology confirmed parasites or only gametocytes without symptoms after recent treatment abroad (n = 24), 2653 episodes were included in the analysis (Figure 1).

Characteristics of patients infected with different species are presented in Table 1. The annual number of malaria episodes increased markedly in 2014 and 2015, explained by high incidence of *P. vivax* in newly arrived Eritrean migrants [16]. Also, the number of severe episodes increased in 2014, due to a high proportion of severe *P. vivax* in this group (Supplementary Figure 1A–C).

**Table 1.** Characteristics of Patients Diagnosed With Malaria in Sweden

Characteristic	All n = 2653^a^	*P. falciparum* n = 1548, 58.3%	*P. vivax* n = 776, 29.2%	*P. ovale* n = 188, 7.1%	*P. malariae* n = 61, 2.3%	Mixed^b^ n = 44, 1.7
Age, y, mean (range)	32.6 (0.2–83)	34.4 (0.2–83)	29.9 (1–79)	30.4 (3–70)	30.2 (3–65)	28.0 (1–68)
Children <18 y, No. (%)	448 (16.9)	220 (14.2)	166 (21.4)	31 (16.5)	11 (18.0)	13 (29.5)
Male gender, No. (%)	1773 (66.8)	1009 (65.2)	536 (69.1)	128 (68.1)	44 (72.1)	33 (75.0)
Patient origin, No. (%)						
Sub-Saharan Africa	1391 (52.4)	902 (58.3)	325 (41.9)	83 (44.2)	37 (60.7)	26 (59.1)
Other endemic^c^	150 (5.6)	21 (1.4)	118 (15.2)	7 (3.7)	3 (4.9)	0 (0)
Nonendemic	1100 (41.5)	616 (39.8)	331 (42.6)	98 (52.1)	20 (32.8)	18 (40.9)
Missing data	12 (0.5)	9 (0.6)	2 (0.3)	0 (0)	1 (1.6)	0 (0)
Region of infection, No. (%)^d^						
Eastern Africa	1125 (42.4)	592 (38.2)	381 (49.1)	86 (45.7)	34 (55.7)	18 (40.9)
Western Africa	878 (33.1)	738 (47.7)	23 (3.0)	71 (37.8)	17 (27.9)	12 (27.3)
Middle Africa	189 (7.1)	135 (8.7)	21 (2.7)	20 (10.6)	6 (9.9)	7 (15.9)
Southern Africa	12 (0.5)	10 (0.6)	0 (0)	2 (1.1)	0 (0)	0 (0)
South Asia	160 (6.0)	14 (0.9)	137 (17.7)	5 (2.7)	3 (4.9)	1 (2.3)
Southeast Asia	140 (5.3)	41 (2.6)	91 (11.7)	0 (0)	1 (1.6)	4 (9.1)
Western Asia	13 (0.5)	2 (0.1)	10 (1.3)	0 (0)	0 (0)	0 (0)
Americas	64 (2.4)	6 (0.4)	56 (7.2)	1 (0.5)	0 (0)	0 (0)
Oceania	52 (2.0)	0 (0)	49 (6.3)	1 (0.5)	0 (0)	2 (4.5)
Europe	2 (0.1)	1 (0.1)^e^	1 (0.1)^f^	0 (0)	0 (0)	0 (0)
Missing data	18 (0.7)	9 (0.6)	7 (0.9)	2 (1.1)	0 (0)	0 (0)
Travelers^g^ length of stay in endemic area, days, median (IQR)	30 (20–60)	30 (16–60)	45 (21–90)	45 (23.5–149)	45 (30–75)	30 (20–90)
Chemoprophylaxis, No. (%)						
Regular	530 (20.0)	282 (18.2)^h^	150 (19.3)	71 (37.8)	11 (18.0)	11 (25.0)
Irregular	349 (13.1)	213 (13.8)	75 (9.6)	33 (17.5)	15 (24.6)	5 (11.4)
No prophylaxis	1674 (63.1)	1003 (64.8)	514 (66.3)	79 (42.0)	34 (55.8)	27 (61.3)
Missing data	100 (3.8)	50 (3.2)	37 (4.8)	5 (2.7)	1 (1.6)	1 (2.3)
Time to symptom onset after return, days, median (IQR)						
Regular chemoprophylaxis	14 (5–90)	7 (2–11)	90 (55–150)	150 (79–210)	72.5 (30–102)	33 (2–63)
Irregular chemoprophylaxis	12 (4–40)	7 (2–14)	60 (28–117.5)	70 (24–120)	31 (14–60)	14 (14–25)
No chemoprophylaxis	7 (1–18)	4 (1–9)	19 (4–87)	54 (8–180)	24 (12–44)	8 (0–30)
Patient delay, days, No. (%)						
0	244 (9.2)	153 (9.9)	64 (8.3)	15 (8.0)	4 (6.6)	1 (2.3)
1–2	701 (26.4)	479 (30.9)	174 (22.4)	31 (16.5)	3 (4.9)	8 (18.2)
3–4	717 (27.0)	462 (29.8)	185 (23.8)	43 (22.9)	12 (19.7)	12 (27.3)
5–6	343 (12.9)	190 (12.3)	101 (13.0)	40 (21.3)	6 (9.8)	5 (11.3)
>6	524 (19.8)	215 (13.9)	206 (26.6)	54 (28.7)	30 (49.2)	11 (25.0)
Missing data	124 (4.7)	49 (3.2)	46 (5.9)	5 (2.6)	6 (9.8)	7 (15.9)

Abbreviation: IQR, interquartile range.

^a^Including 1 case of *P. knowlesi*, 23 cases with gametocytes only, and 12 cases with unknown species.

^b^Including *P. falciparum* in combination with *P. vivax* (20), *P. ovale* (9) and *P. malariae* (9); *P. vivax* in combination with *P. ovale* (1) and *P. malariae* (1)*; P. ovale* in combination with *P. malariae* (1); *P. falciparum* gametocytes and *P. vivax* (2) and *P. ovale* (1).

^c^Caribbean (2), Central America (1), South America (7), South Asia (107), Southeast Asia (7), Western Asia (19), Oceania (7).

^d^According to UN Geoscheme.

^e^This patient denied any travel to malaria endemic areas.

^f^This patient became ill after long transit through Turkey.

^g^In travelers residing in Sweden before traveling.

^h^219/282 (77.7%) in patients on chloroquine with or without addition of proguanil.

### Clinical Presentation

Severe malaria defined according to the WHO 2015 criteria was present in 193/2653 (7.3%) patients, and 34 patients deteriorated after initiated treatment rendering 227/2653 (8.6%) severe episodes. Severe malaria was seen in *P. falciparum* (146/1548, 9.4%), *P. vivax* (60/776, 7.7%), *P. ovale* (10/188, 5.3%), *P. malariae* (2/61, 3.3%), and in mixed *P. falciparum* episodes (8/38, 21.1%) (Table 2). One case of nonsevere *P. knowlesi* was diagnosed using polymerase chain reaction (PCR). The cases deteriorating after initiated treatment were caused by *P. falciparum* (n = 25), *P. vivax* (n = 7), and unknown species (n = 1).

**Table 2.** Severe Malaria Criteria in Episodes of Different Species (n=227)^a^

Criteria	*P. falciparum* n = 146/1548, 9.4%	*P. vivax* n = 60/776, 7.7%	*P. ovale* n = 10/188, 5.3%	*P. malariae* n = 2/61, 3.3%	Mixed^b^ n = 8/44, 18.2%
WHO criteria					
Impaired consciousness	28	1	1	0	1
Prostration	9	1	0	0	0
Multiple convulsions	9	0	0	0	0
Acidosis	21	0	0	0	0
Hypoglycemia	2	0	0	0	0
Severe anemia	16^c^	15	0	1	4
Renal impairment	31	1	0	0	3
Hyperbilirubinemia	66^c^	28	6	0	4
Pulmonary edema	15	4	2	0	0
Significant bleeding	17	5	1	0	1
Shock	33	8	2	1	3
Hyperparasitemia	57	0	0	0	3
Numbers of criteria fulfilled per episode of severe malaria, No. (%)^d^					
1	79 (59.4)	57 (95.0)	8 (80)	2 (100)	4 (50.0)
2	25 (18.8)	3 (5.0)^e^	2 (20)^f^	0 (0)	1 (12.5)
3	15 (11.3)	0 (0)	0 (0)	0 (0)	0 (0)
4	5 (3.7)	0 (0)	0 (0)	0 (0)	2 (25.0)
≥5	9 (6.8)	0 (0)	0 (0)	0 (0)	1 (12.5)
Criteria prognostic of unfavorable outcome, No. (% of all cases)^g^	84 (5.4)	18 (2.3)	4 (2.1)	1 (1.6)	5 (11.4)
Fatal outcome, No. (% of the severe)	3 (2.1)	0 (0)	0 (0)	0 (0)	1 (12.5)^h^

^a^One episode of severe malaria with unknown species fulfilled the criteria for circulatory shock.

^b^Of the mixed infections including *P. falciparum*, 8/38 (21.1%) were severe, thus all severe episodes with mixed *Plasmodium* included *P. falciparum*.

^c^In *P. falciparum*, severe anemia and hyperbilirubinemia includes parasitemia thresholds.

^d^Hyperparasitemia is excluded in this comparison.

^e^Consisting of: prostration and shock (1), bleeding and shock (1), and hyperbilirubinemia and pulmonary edema (1).

^f^Consisting of impaired consciousness and shock (1), pulmonary edema and shock (1).

^g^Including coma, multiple convulsions, acidosis, renal impairment, shock, pulmonary edema, and significant bleeding.

^h^ *P. falciparum* and *P. ovale*.

The dominating criteria for severe *P. vivax* were hyperbilirubinemia (28/60, 46.7%) and severe anemia (15/60, 25.0%). Also, in severe *P. falciparum,* hyperbilirubinemia with the >2% parasitemia threshold, was the most common single WHO criterion (27/79, 34.2%) (Table 2). Combinations of ≥2 criteria were present in 54/133 (40.6%) severe *P. falciparum* (not including hyperparasitemia), whereas only in 5/72 (6.9%) severe non-*falciparum* episodes (*P* < .001) (Table 2). Criteria prognostic of unfavorable outcome were most common in *P. falciparum* (84/1548, 5.4%) but were also reported in non-*falciparum* episodes (23/1025, 2.2%) (*P* < .001), corresponding to 84/146 (57.5%) of severe *P. falciparum* and 23/72 (31.9%) of the severe non-*falciparum* cases, respectively (*P* < .001).

In *P. falciparum,* severe malaria was associated with age 0–5 years (adjusted odds ratio [aOR], 4.4 [95% confidence interval, CI, 1.7–11.6]; *P* = .003) and >60 years (aOR, 5.5 [95% CI, 2.5–11.7]; *P* < .001), respectively, using age group 18–29 years as reference and adjusting for patient origin (nonendemic, sub-Saharan Africa, and other endemic), region of diagnosis (Stockholm and Gothenburg compared to remaining clinics), and health care delay (Table 3). Female or male sex did not affect the outcome and was therefore not included.

**Table 3.** Characteristics of Patients With Nonsevere and Severe *P. falciparum* and Non-*falciparum* Malaria

Characteristic	*P. falciparum* ^*a*^					Non-*falciparum*^a^				
	No. of patients (%)		*P* Value	Unadjusted OR (95% CI)	Adjusted^b^ OR (95% CI)	No. of patients (%)		*P* Value	Unadjusted OR (95% CI)	Adjusted^c^ OR (95% CI)
	Nonsevere, n = 1402	Severe, n = 146				Nonsevere n = 953	Severe n = 72			
Age, y, median (range)	34.0 (0.2–83)	40.5 (0.7–75)	.002^d^			27.0 (1–79)	24.5 (14–69)	.58^d^		
Age group, y										
0–5	39 (79.6)	10 (20.4)	<.001^e^	5.5 (2.3–13.2)	4.4 (1.7–11.6)	22 (100)	0 (0)	.015^f^	No cases	No cases
6–17	154 (90.1)	17 (9.9)		2.2 (1.1–4.7)	2.6 (1.2–5.4)	173 (93.0)	13 (7.0)		0.7 (.4–1.4)	0.6 (.3–1.2)
18–29	336 (94.6)	19 (5.4)		1 (ref)	1 (ref)	355 (90.6)	37 (9.4)		1 (ref)	1 (ref)
30–39	382 (93.9)	25 (6.1)		1.1 (.5–2.1)	1.3 (.6–2.6)	171 (95.5)	8 (4.5)		0.45 (.2–1.0)	0.4 (.2–.8)
40–49	262 (90.7)	27 (9.3)		2.2 (1.2–4.3)	2.6 (1.3–5.1)	116 (98.3)	2 (1.7)		0.1 (.01–.6)	0.1 (.01–.8)
50–59	167 (86.5)	26 (13.5)		3.2 (1.7–6.3)	3.0 (1.5–5.9)	78 (92.9)	6 (7.1)		0.7 (.3–1.8)	0.9 (.3–2.1)
≥60	62 (73.8)	22 (26.2)		7.4 (3.6–15.2)	5.5 (2.6–11.7)	38 (86.4)	6 (13.6)		1.4 (.6–3.7)	1.7 (.7–4.6)
Female gender	486 (90.2)	53 (9.8)	.715^f^	1 (ref)	1 (ref)	301 (95.0)	16 (5.0)	.113^f^	1 (ref)	1 (ref)
Male gender	916 (90.8)	93 (9.2)		1.1 (.8–1.7)	1.0 (.7–1.5)	652 (92.1)	56 (7.9)		1.6 (.9–3.0)	1.5 (.8–2.7)
Patient origin										
Nonendemic	532 (86.4)	84 (13.6)	<.001^f^	2.6 (1.8–3.8)	2.1 (1.4–3.1)	425 (94.6)	24 (5.4)	.037^f^	0.5 (.3–.9)	0.5 (.3–.9)
Sub-Saharan Africa	841 (93.2)	61 (6.8)		1 (ref)	1 (ref)	403 (90.6)	42 (9.4)		1 (ref)	1 (ref)
Other endemic	20 (95.2)	1 (4.8)		0.8 (.1–6.4)	0.8 (.1–5.0)	122 (95.3)	6 (4.7)		0.5 (.2–1.2)	0.6 (.2–1.5)
Missing	9 (100)	0 (0)				3 (100)	0 (0)			
Chemoprophylaxis										
Regular	257 (91.1)	25 (8.9)	.184^f^	1 (ref)	1 (ref)	223 (92.1)	9 (3.9)	.029^f^	1 (ref)	1 (ref)
Irregular	199 (93.4)	14 (6.6)		0.7 (.4–1.5)	1.1 (.5–2.3)	116 (94.3)	7 (5.7)		1.3 (.4–3.7)	1.5 (.5–4.2)
No prophylaxis	897 (89.4)	106 (10.6)		1.2 (.4–1.5)	2.1 (1.3–3.6)	571 (91.1)	56 (8.9)		2.5 (1.2–5.1)	2.7 (1.1–6.3)
Missing data	49 (98.0)	1 (2.0)				43 (100)	0 (0)			
Years lived in nonendemic country, in patients with origin in malaria endemic countries	Patients of Sub-Saharan Africa origin only, n = 841	n = 61				Patients originating in malaria endemic countries, n = 525	n = 48			
<1	208 (89.3)	25 (10.7)	.021^f^	3.7 (1.2–11.0)	2.8 (.8–9.7)	360 (89.8)	41 (10.2)	.082^f^	1 (ref)	1 (ref)
1–2	50 (100)	0 (0.0)		No cases	No cases	19 (86.4)	3 (13.6)		1.4 (.4–4.9)	1.6 (.5–7.3)
3–4	80 (95.2)	4 (4.8)		2.1 (.5–8.5)	2.3 (.5–9.5)	14 (100)	0 (0)		No cases	No cases
5–9	160 (96.4)	6 (3.6)		1 (ref)	1 (ref)	23 (95.8)	1 (4.2)		0.4 (.05–2.9)	0.5 (.1–2.3)
10–14	121 (93.8)	8 (6.2)		2.0 (.6–7.3)	1.9 (.5–7.1)	32 (100)	0 (0)		No cases	No cases
≥15	148 (91.4)	14 (8.6)		3.3 (1.1–10.6)	2.7 (.8–8.9)	28 (100)	0 (0)		No cases	No cases
Missing data	74 (94.9)	4 (5.1)				33 (94.3)	2 (5.7)			
HIV positive	23 (76.7)	7 (23.3)	.173^f^	2.1 (.9–5.3)	3.4 (1.2–10.3)	7 (87.5)	1 (12.5)	.443^f^	1.9 (.2–15.5)	2.3 (.2–32.3)
Pregnancy	17 (68.0)	8 (32.0)	<.001^f^	7.4 (3.0–18.2)	5.7 (1.9–16.4)	13 (100)	0 (0.0)	1.0	No cases	No cases
Place of diagnosis										
Stockholm or Gothenburg	824 (93.5)	57 (6.5)	<.001^d^	1 (ref)	1 (ref)	387 (93.7)	26 (6.3)	.453^d^	1 (ref)	1 (ref)
Other	578 (86.7)	89 (13.3)		2.1 (1.5–3.1)	2.0 (1.4–2.9)	566 (92.5)	46 (7.5)		1.3 (.8–2.1)	1.1 (.6–1.8)
Patient delay, days										
0	140 (91.5)	13 (8.5)	.009^f^	1 (ref)	1(ref)	80 (96.4)	3 (3.6)	.282^f^	1 (ref)	1 (ref)
1–2	451 (94.2)	28 (5.8)		0.6 (.3–1.3)	0.8 (.4–1.7)	197 (94.7)	11 (5.3)		1.5 (.4–5.6)	1.5 (.4–5.7)
3–4	405 (87.7)	57 (12.3)		1.5 (.8–2.8)	2.0 (1.0–4.0)	223 (92.9)	17 (7.1)		1.9 (.6–6.9)	2.2 (.6–7.9)
5–6	168 (88.4)	22 (11.6)		1.5 (.7–3.1)	2.1 (.0–4.6)	137 (93.2)	10 (6.8)		2.0 (.5–7.3)	2.1 (.5–8.4)
>6	195 (90.7)	20 (9.3)		1.1 (.2–3.2)	1.4 (.6–3.1)	262 (90.3)	28 (9.7)		3.0 (.9–10.0)	3.5 (1.0–11.8)
Missing data	43 (87.8)	6 (12.2)				54 (94.7)	3 (5.3)			
Health care delay, days										
0	1185 (92.0)	103 (8.0)	<.001^f^	1 (ref)	1 (ref)	736 (93.2)	54 (6.8)	.118^f^	1 (ref)	1 (ref)
1–2	97 (78.2)	27 (21.8)		3.1 (1.9–5.1)	3.4 (1.9–5.9)	83 (95.4)	4 (4.6)		0.5 (.1–1.6)	0.6 (.2–1.9)
3–4	30 (79.0)	8 (21.0)		3.3 (1.5–7.4)	3.3 (1.4–7.7)	48 (85.7)	8 (14.3)		2.3 (1.0–5.2)	2.8 (1.1–6.7)
5–6	16 (72.7)	6 (27.3)		3.9 (1.4–10.8)	3.8 (1.2–11.6)	18 (85.7)	3 (14.3)		2.2 (.6–7.8)	2.9 (.8–10.1)
>6	25 (92.6)	2 (7.4)		1.0 (.2–4.4)	0.7 (.2–3.1)	44 (95.7)	2 (4.3)		0.6 (.1–2.6)	0.7 (.2–3.1)
Days admitted to hospital, median (IQR)	2 (1–3)	7 (4–11)	<.001^d^			2 (0–3)	3 (2–4)	<.001^d^		

Abbreviations: CI, confidence interval; IQR, interquartile range; OR, odds ratio.

^a^Includes monoinfections only.

^b^Adjusted for age group, patient origin, region of diagnosis, and health care delay. Episodes with confirmed pregnancy or HIV infection were excluded due to risk of confounding, except where otherwise stated.

^c^Adjusted for age group, patient origin, and health care delay.

^d^Mann-Whitney U test.

^e^ *Χ* ^2^ test.

^f^Fisher exact test.

Pregnancy was strongly associated with severe *P. falciparum* malaria (OR, 7.4 [95% CI, 3.0–18.2]; *P* < .001); however, including pregnancy in the multivariate model caused instability and a drift in ORs likely to be explained by the small sample size. Similar effect on model stability was observed for HIV positivity, therefore, to avoid confounding, all episodes with confirmed pregnancy (n = 39) or HIV infection (n = 38) were excluded from the multivariate analysis.

Origin in Sweden or other nonendemic countries was associated with severe *P. falciparum* (aOR, 2.1 [95% CI, 1.4–3.1]); also regarding ≥2 criteria (aOR, 4.4 [95% CI, 2.3–8.7]), and criteria prognostic of unfavorable outcome (aOR, 3.2 [95% CI, 1.8–5.7]) (all *P* < .001) (Table 3).

Newly arrived migrants (<1 year in a nonendemic country) and immigrants living in Sweden or another nonendemic country for ≥15 years had an increased odds of severe *P. falciparum* malaria; however, this was nonsignificant after adjustments for age group, region of presentation, and health care delay (aOR, 2.8 [95% CI, .8–9.7]; *P* = .10 and aOR, 2.7 [95% CI, .8–8.9]; *P* = .10, respectively) (Table 3).

Severe non-*falciparum* (*P. vivax, P. ovale* and *P. malariae*) was associated with origin in sub-Saharan Africa (aOR, 2.0 [95% CI, 1.1–3.4]; *P* = .015) adjusting for age group and health care delay. Of all severe non-*falciparum* episodes, 42/72 (58.3%) were in patients originating from sub-Saharan Africa, and in this group most (41/42, 97.6%) were recently arrived migrants, most commonly from Eritrea (32/42, 76.2%) diagnosed in 2014–2015.

### Management

Diagnosis were delayed ≥1 day after health care presentation in 211/1499 (14.1%) *P. falciparum* and 210/1000 (21.0%) non-*falciparum* episodes (Table 4). Health care delay was more common in children ≤5 years (25/71, 35.2%) than in older children and adults (412/2502, 16.5%) (*P* < .001), and more common in patients born in Sweden or other nonendemic countries (193/1057, 18.3%) compared to patients born in sub-Saharan Africa (195/1361, 14.3%) (*P* = .010), and also in females (162/846, 19.2%) compared to males (275/1727, 15.9%) (*P* = .044).

**Table 4.** Management, Treatments, and Outcome of Malaria Caused by Different Species

Characteristic	All n = 2653	*P. falciparum* n = 1548	*P. vivax* n = 776	*P. ovale* n = 188	*P. malariae* n = 61	Mixed n = 44
Health care delay, days, No. (%)^a^						
0	2136 (80.5)	1288 (83.2)	607 (78.2)	139 (73.9)	44 (72.2)	36 (81.8)
1–2	217 (8.2)	124 (8.0)	62 (8.0)	22 (11.7)	3 (4.9)	3 (6.8)
3–4	98 (3.7)	38 (2.5)	39 (5.0)	14 (7.5)	3 (4.9)	1 (2.3)
5–6	43 (1.6)	22 (1.4)	16 (2.1)	4 (2.1)	1 (1.6)	0 (0)
>6	79 (3.0)	27 (1.7)	33 (4.3)	4 (2.1)	9 (14.8)	4 (9.1)
Missing data	80 (3.0)	49 (3.2)	19 (2.4)	5 (2.7)	1 (1.6)	0 (0)
Days from symptom onset to initiation of treatment, median (IQR)	4 (2–7)	3 (2–5)	4 (2–7)	6 (4–8)	7 (5–14)	5 (2–14)
Admitted to hospital, No. (%)	2169 (81.8)	1342 (86.7)	595 (76.7)	132 (70.2)	40 (65.6)	40 (90.9)
Days admitted to hospital, median (IQR)	2 (1–4)	3 (1–4)	2 (1–3)	1 (0–3)	1 (0–3)	2 (1.5–4.5)
Admitted to ICU, No. (%)	127 (4.8)	110 (7.1)	10 (1.3)	1 (0.5)	1 (1.6)	5 (11.4)
Days admitted to ICU, median (IQR)	2 (1–4)	2 (1–5)	1 (1–2)	2 (…)	1 (…)	3 (1–9)
First time relapse or recrudescence, No. (%)	98 (3.7)	12 (0.8)	77 (9.9)	5 (2.7)	1 (1.6)	3 (6.8)^b^
Intravenous antimalarial treatment, No. (%)^c^	352 (13.3)	312 (20.2)	21 (2.7)	5 (2.7)	5 (8.2)	8 (18.2)
Intravenous antimalarial treatment in severe malaria (n = 227), No. (%)^c^	110/227 (48.5)	103/146 (70.6)	4/60 (6.7)	0/10 (0)	0/2 (0)	3/8 (37.5)
Intravenous antimalarial treatment in nonsevere malaria (n = 2426), No. (%)	242 (10.0)	209 (14.9)	17 (2.4)	5 (2.8)	5 (8.5)	5 (13.9)
Blood transfusion, No. (%)	112 (4.3)	81 (5.3)	21 (2.7)	1 (0.5)	1 (1.6)	6 (13.6)
Blood exchange transfusion, No. (%)^d^	15 (0.6)	14 (0.9)	0 (0)	0 (0)	0 (0)	1 (2.3)

Abbreviations: ICU, intensive care unit; IQR, interquartile range.

^a^Delayed treatment ≥1 day after health care presentation.

^b^Consisting of 1 *P. falciparum* recrudescence, 1 *P. vivax* relapse, and 1 *P. malariae* relapse.

^c^Initiated at arrival or during hospital stay.

^d^All were performed in severe malaria patients and none after year 2007.

In *P. falciparum,* the odds of severe malaria increased when diagnosis and treatment were delayed (Table 3). Likewise, health care delay was associated with increased odds for severe malaria with criteria prognostic of unfavorable outcome: 1–2 days (aOR, 4.4 [95% CI, 2.2–8.8]; *P* < .001), 3–4 days (aOR, 4.0 [95% CI, 1.4–11.5]; *P* = .01) and 5–6 days of delay (aOR, 3.8 [95% CI, 1.1–13.9]; *P* = .041).

Malaria diagnosis in city or region other than the largest 2 cities in Sweden, Stockholm and Gothenburg, where half of the cases 1331/2653 (50.2%) were diagnosed, was associated with severe *P. falciparum* malaria (aOR, 2.0 [95% CI, 1.4–2.9]; *P* < .001), adjusting for age group, patient origin, and health care delay. Adjusting for patient delay did not contribute to the model (*P* = .71) or affect the outcome.

In non-*falciparum*, odds for severe malaria were not increased for 1–2 days of health care delay (aOR, 0.6 [95% CI, .2–1.9]; *P* = .35), but were nearly 3 times higher in cases with 3–4 days of health care delay (aOR, 2.8 [95% CI, 1.1–6.7]; *P* = .024) and 5–6 days (aOR, 2.9 [95% CI, .8–10.1]; *P* = .09), after adjusting for age group and patient origin (Table 3).

Most patients were admitted to hospital (2169/2653, 81.8%) and 127/2653 (4.8%) were admitted to an ICU (Table 4). Among all ICU-admitted patients, 92/127 (72.4%) fulfilled at least 1 WHO criterion for severe malaria. Patients with severe *P. falciparum* and ≥2 criteria were more often admitted to ICU (60/72, 83.3%) compared to 1 criterion (22/74, 29.7%), and similarly in episodes with and without criteria prognostic of unfavorable outcome (64/84, 76.2% vs 18/62, 29.0%) (all *P* < .001). Admission to hospital was longer in severe *P. falciparum* episodes fulfilling criteria prognostic of unfavorable outcome compared to other severe *P. falciparum* episodes (median 8 days, IQR 6–15 vs 5 days, IQR 4–7, *P* = .0023).

Among the severe non-*falciparum* episodes, 7/72 (9.7%) were admitted to ICU, and all 7 fulfilled criteria prognostic of unfavorable outcome. In the severe episodes not requiring ICU care, hyperbilirubinemia alone was the most common criterion in both *P. falciparum* (22/64, 34.4%) and non-*falciparum* (33/67, 49.3%). Management is further described in Table 4.

Blood cultures were obtained in 1077/2653 (40.6%) episodes and 89/146 (61.0%) severe *P. falciparum* episodes. Significant bacterial growth was found in 8/1077 (0.7%) and 3/89 (3.4%) in severe *P. falciparum* (*Acinetobacter baumannii, Staphylococcus aureus,* and micrococcus species). In severe non-*falciparum,* blood cultures were obtained from 44/72 (61.1%) patients; none had a significant bacteremia.

### Outcome

In total, 4 deaths occurred in the study population, corresponding to an overall case fatality rate (CFR) of 0.15% (4/2653); 0.25% (4/1582) for *P. falciparum* and 2.6% (4/154) for severe *P*. *falciparum*. All were male travelers born in Sweden, resulting in a CFR of 1.7% (4/239) in nonendemic-born males and 7.4% (4/54) in those with severe *P. falciparum*. Three cases were treated with quinine and in 1 case, combination of artesunate and quinine. No deaths occurred in the study population between 2011 and 2015.

Progression to severe malaria during treatment occurred in 25/1548 (1.6%) of the initially nonsevere *P. falciparum* episodes and in 8/1025 (0.8%) of the non-*falciparum* episodes (*P* = .066), respectively. Of these, 16/25 (64.0%) *P. falciparum* and 5/8 (62.5%) non-*falciparum* episodes progressed to severe malaria with criteria prognostic of unfavorable outcome.

Parasitemia, only reported in *P. falciparum*, ranged between 0.002% and 8.4% in the nonsevere *P. falciparum* episodes started on oral treatment, with median parasitemia ranging from 0.1% to 0.5% among the different oral regiments (*P* = .25). *P. falciparum* episodes with parasitemia >2% at presentation and initial oral treatment had an increased odds for progression to severe malaria with criteria prognostic of unfavorable outcome (OR, 8.7 [95% CI, 1.9–39.3]; *P* = .005), occurring during treatment with atovaquone/proguanil (n = 1/27), mefloquine (n = 2/68), oral quinine (n = 2/10), and artemether/lumefantrine (n = 3/43).

## DISCUSSION

This study summarizes the clinical presentation, management, and outcome of imported malaria, using nearly complete nationwide data in Sweden over 20 years. Severe malaria caused by both *P. falciparum* and non-*falciparum* species was identified using the WHO criteria. The most severe cases fulfilling criteria prognostic of unfavorable outcome were more commonly reported in *P. falciparum* episodes but were also found among the non-*falciparum* episodes. Factors associated with severe *P. falciparum* malaria were age, pregnancy, HIV infection, origin in nonendemic country, health care delay, and presentation in a region where malaria is less often diagnosed, as previously described [7, 8, 10, 13, 17–19]. In addition, we identified newly arrived migrants to be a risk group for severe malaria. Moreover, we identified older age and longer health care delay as risk factors for severe non-*falciparum* malaria.

In *P. falciparum*, these risk factors predicted also the most severe forms of malaria, reflected by presence of criteria prognostic of unfavorable outcome. Nonetheless, the current WHO criteria for severe malaria (2015) [1] did not always reflect disease severity considering the low proportion of ICU admission in severe episodes without criteria prognostic of unfavorable outcome compared to those fulfilling these criteria (29.0% vs 76.2%). Our findings suggest that the criteria for severe malaria need to be optimized to different parasite species to better guide the management of imported malaria.

Severe malaria is well recognized to be caused predominantly by *P. falciparum*, although severe forms are described in all species [3, 4, 20, 21]. Severe episodes in *P. ovale* and *P. malariae* are more rare [4, 22], and not even included in the WHO criteria for severe malaria [1]. Here, most severe and all fatal episodes were caused by *P. falciparum*. Nonetheless, the proportion of severe *P. vivax* fulfilling the WHO criteria but also criteria for poor prognosis was notable, especially among newly arrived Eritreans arriving 2014 and 2015. A smaller subset of the severe episodes was caused by *P. ovale* and *P. malariae* (using the severe *P. vivax* criteria), some also fulfilled criteria prognostic of unfavorable outcome and were admitted to ICU, of which there have been only scarce previous reports [4, 22].

Furthermore, severe malaria was more common in mixed *P. falciparum* infections than in *P. falciparum* monoinfections. The effect of simultaneous presence of another species of *Plasmodium* is not fully elucidated [23–25]; here, our results support an increased risk of severe malaria in mixed infections, including 1 case of death, although the most common criteria were anemia and hyperbilirubinemia.

In severe *P. vivax,* both multiorgan failure and death have been reported in endemic areas [23, 24]. Among the imported *P. vivax* episodes in Sweden, we could not see the same level of severity and most episodes fulfilled only 1 criterion for severe *P. vivax* malaria, dominated by severe anemia or hyperbilirubinemia. Severe anemia could be debilitating and might require blood transfusions. Hyperbilirubinemia has been described as a common feature of *P. falciparum* and *P. vivax* malaria, appearing as a sign of erythrocyte destruction or liver dysfunction [25]. However, hyperbilirubinemia as a single criterion for severe malaria does not seem to have a convincing impact on severity or mortality [15, 17, 26, 27]. This is supported by the findings in our study where hyperbilirubinemia irrespective of species often appeared without other signs of complicated disease, thus it does not seem to be a suitable criterion for imported severe malaria. In contrast, the selected criteria prognostic of unfavorable outcome (impaired consciousness, multiple convulsions, acidosis, renal impairment, shock, pulmonary edema, and significant bleeding) [14, 15] were strongly associated with ICU admission.

The case fatality rate in our study corresponds to previous studies in nonendemic settings [8, 20], and was particularly high (7%) among nonimmune male travelers with severe *P. falciparum* malaria. The overall malaria mortality in Sweden is, however, not known and our data only reflect the diagnosed cases. A study in the United States suggested that 15% of the lethal malaria cases were diagnosed at autopsy [8, 28].

Delayed diagnosis and antimalarial treatment are well-known risk factors for severe malaria and death [8, 10]. Our analysis showed that diagnosis was delayed at least 1 day after health care presentation in nearly one-fifth of all malaria episodes, as previously observed in other nonendemic settings [29]. For *P. falciparum,* health care delay was associated with severe malaria, criteria prognostic of unfavorable outcome, and ICU admission. Even in non-*falciparum* episodes, health care delay of 3 days or more affected disease severity significantly. Health care delay was most common in young children and patients born in nonendemic countries. This further emphasizes the necessity to enquire about travel history and promptly investigate for malaria in febrile patients returning from malaria-endemic countries, which is a challenge in a country such as Sweden where malaria is a relatively rare disease.

Health care presentation outside the 2 main cities in Sweden, Stockholm and Gothenburg, was an independent risk factor for severe *P. falciparum* malaria. This effect remained after adjusting for age, patient origin, health care delay, and even patient delay. Because most severe episodes fulfilled WHO criteria already at hospital admission, differences in the management of malaria in hospitals is unlikely to explain this effect. Rather, some differences in health care delay not mentioned in the medical chart may have contributed to the higher morbidity.

Episodes of *P. falciparum* progressing to severe malaria during oral treatment had a higher parasitemia compared to episodes without deterioration. Even though the number of episodes progressing to severe malaria is small, our findings suggest that high parasitemia is a warning sign, also acknowledged in the previous WHO 2010 definition of severe malaria where parasitemia >2% was specified as a criterion for severe malaria in nonimmune individuals and >5% in the expected immune [30]. In the current WHO 2015 definition [1], hyperparasitemia >10% is defined as single criterion; hence, patients without other severe signs and parasitemia <10% are recommended oral treatment. Our findings support early consideration of intravenous treatment at parasitemia ≥2% even in absence of other severe signs, as reported in previous studies [14, 31] and stated in the present United Kingdom treatment guidelines [32].

Although patients born in malaria-endemic countries account for a proportionally high number of the imported *P. falciparum* cases, severe malaria and death is generally recognized to be less frequent in this group [8, 33–35], influenced by immunity acquired from previous infections [36]. However, protective immunity appears to decline without continuous exposure [37, 38]. Here, the odds of severe *P. falciparum* among African immigrants increased with time of residency in Sweden, confirming our previous findings within a subset of Stockholm patients [37]. Interestingly, the odds of severe *P. falciparum* and non-*falciparum* episodes in newly arrived migrants from sub-Saharan Africa were even higher. This was most pronounced in non-*falciparum,* where over 50% of the severe episodes occurred in newly arrived migrants, possibly caused by prolonged symptom duration or insufficient treatment for prevention of relapse infections as well as factors associated with migration, including poor health care, malnutrition, and stress [23, 39–41]. Within this group, the fulfilled severe criteria were mainly hyperbilirubinemia and severe anemia, but also the most severe signs were recorded. Hence, a reduced risk of severe malaria cannot be presumed in the management of imported malaria in patients of African origin, and both newly arrived migrants and immigrants with long residency should be considered as potential risk groups for more severe malaria.

The strength of this study is the large dataset and nationwide coverage, containing detailed clinical data from the majority of malaria episodes reported to the national surveillance system during 2 decades. The limitations are inherent in the retrospective design and data relying on medical records with varying level of detail. Moreover, neither single nor mixed infections were systematically confirmed by PCR, thus underdiagnosis of mixed infections with *P. falciparum* may have contributed to the high rates of severe malaria observed in non-*falciparum* infections [42].

Our study contributes to the understanding of severity of imported malaria in different species and patient categories. Further studies are needed to establish effective severity criteria, adjusted to different species, populations, and settings that in turn could contribute to an improved management of malaria. Moreover, the findings of increased risk of severe malaria irrespective of species in newly arrived migrants highlights the need for increased attention to malaria in migrants.

## CONCLUSION

In this nationwide clinical assessment of imported malaria in Sweden over 2 decades, we observed severe malaria, also with the most severe signs, caused by both *P. falciparum* and non-*falciparum* species. Moreover, we identified newly arrived migrants as a new risk group for severe malaria. The WHO criteria for severe *P. falciparum* and non-*falciparum* malaria did not effectively reflect disease severity in this nonendemic setting, and improved criteria are needed to better guide the management of imported malaria in travelers and migrants.

## SUPPLEMENTARY DATA

Supplementary materials are available at *The Journal of Infectious Diseases* online. Consisting of data provided by the authors to benefit the reader, the posted materials are not copyedited and are the sole responsibility of the authors, so questions or comments should be addressed to the corresponding author.

jiz292_suppl_Supplementary_Figure_1a

jiz292_suppl_Supplementary_Figure_1b

jiz292_suppl_Supplementary_Figure_1c

jiz292_suppl_supplementary_Table_1

jiz292_suppl_Supplementary_Figure_Legend

## Notes

***Acknowledgments.*** We thank all departments of infectious diseases, departments of pediatrics, and other departments for contributing with medical records; and Ulf Hammar, presently at the Department of Medical Sciences, Uppsala University, for his input on data analysis.

***Financial support.*** This work was supported by Swedish Research Council (grant numbers 348-2013-6573 and 2015-09277); Stockholm County Council (grant numbers 20130207 and 20150135); and Centre for Clinical Research, Västmanland County Council (grant number LTV-633331).

***Potential conflicts of interest.*** All authors: No reported conflicts of interest. All authors have submitted the ICMJE Form for Disclosure of Potential Conflicts of Interest. Conflicts that the editors consider relevant to the content of the manuscript have been disclosed.

Presented in part: 7th North European Conference of Travel Medicine, Stockholm, Sweden, 2–4 May 2018.
