# Supplementary figures and images for: Severity of Plasmodium falciparum and Non-falciparum Malaria in Travelers and Migrants: A Nationwide Observational Study Over 2 Decades in Sweden
Source: J Infect Dis. 2019 Jun 6;220(8):1335–45. doi: 10.1093/infdis/jiz292 (PMC6743839; doi:10.1093/infdis/jiz292)

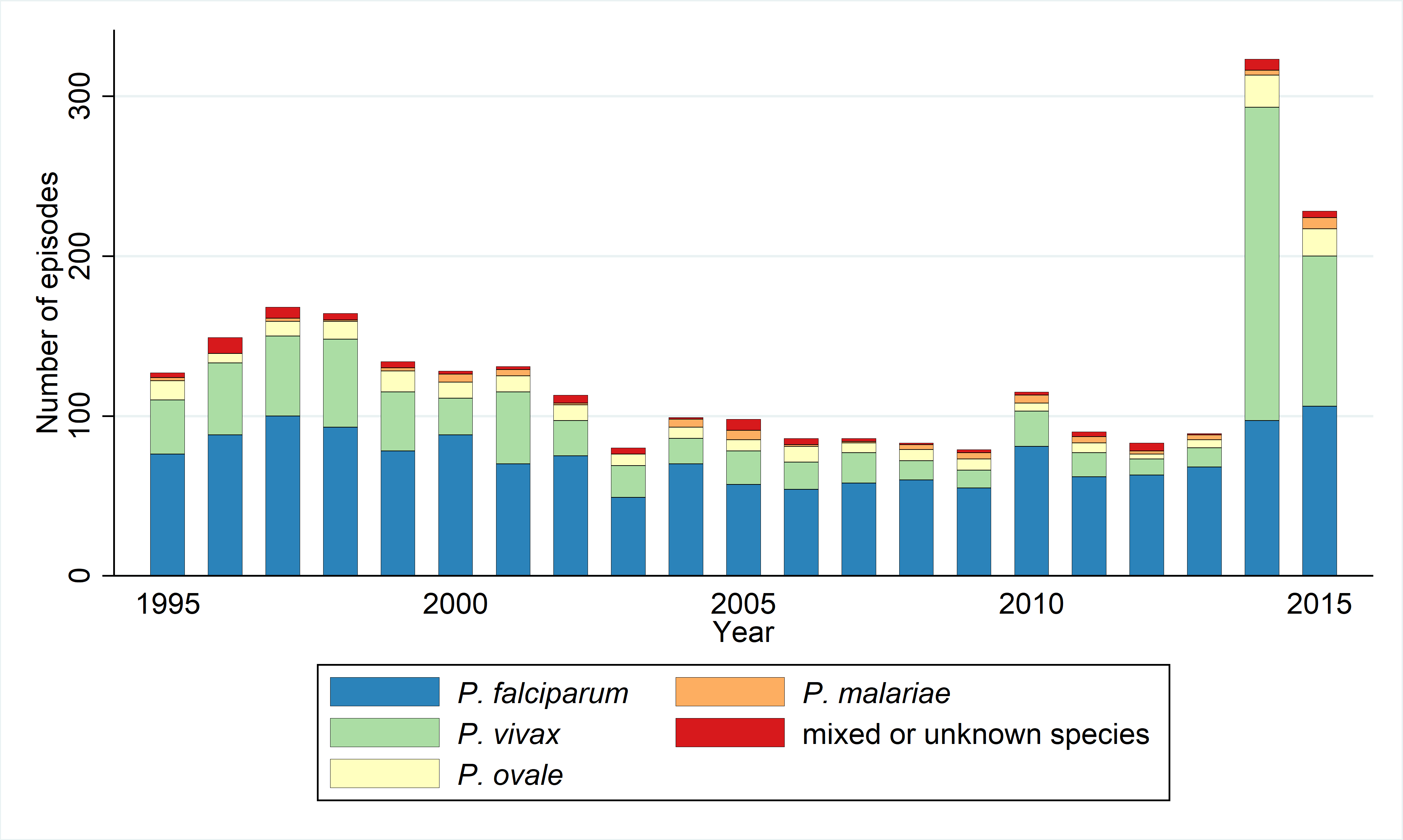

Supplement: jiz292_suppl_Supplementary_Figure_1a [file jiz292_suppl_supplementary_figure_1a.png]

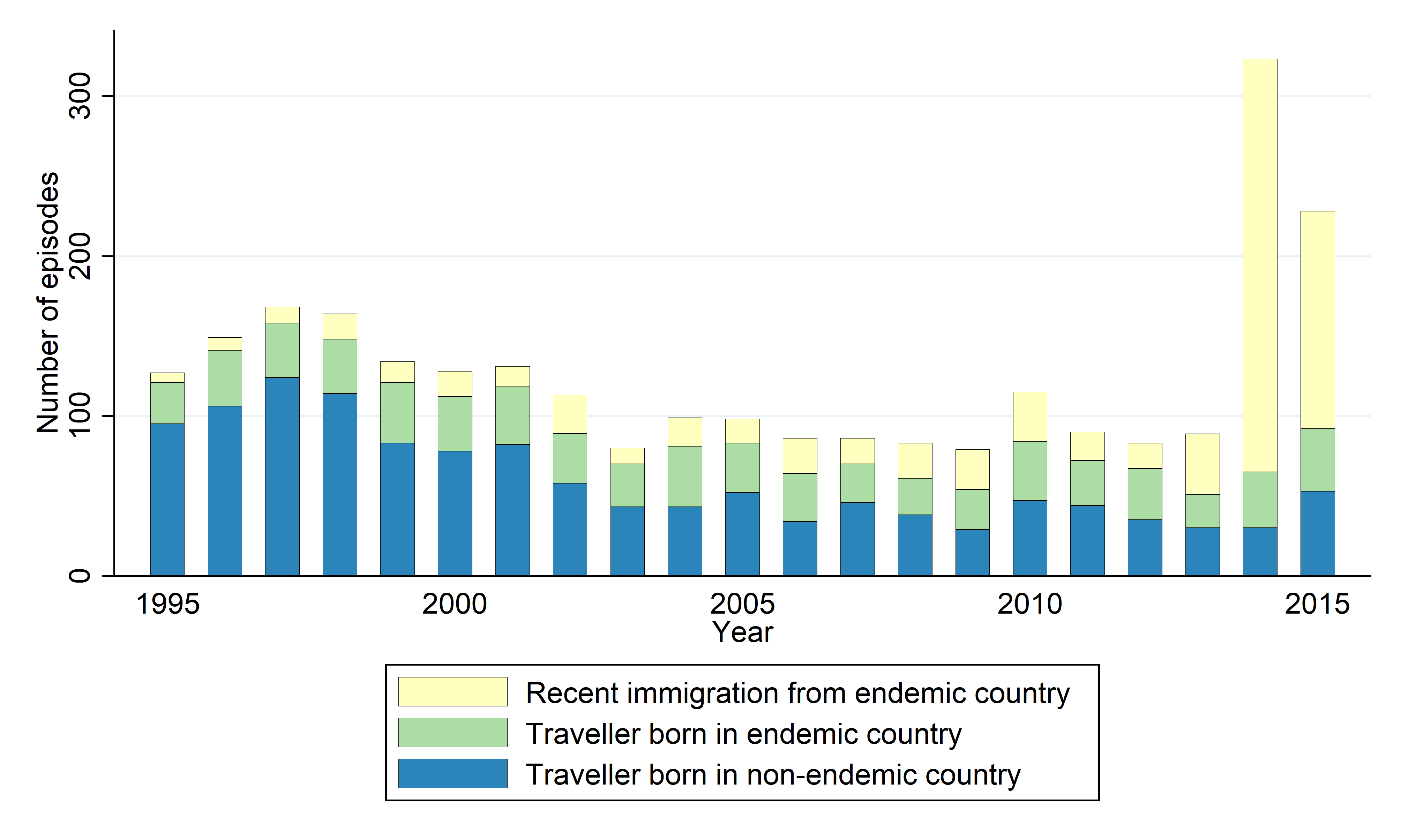

Supplement: jiz292_suppl_Supplementary_Figure_1b [file jiz292_suppl_supplementary_figure_1b.png]

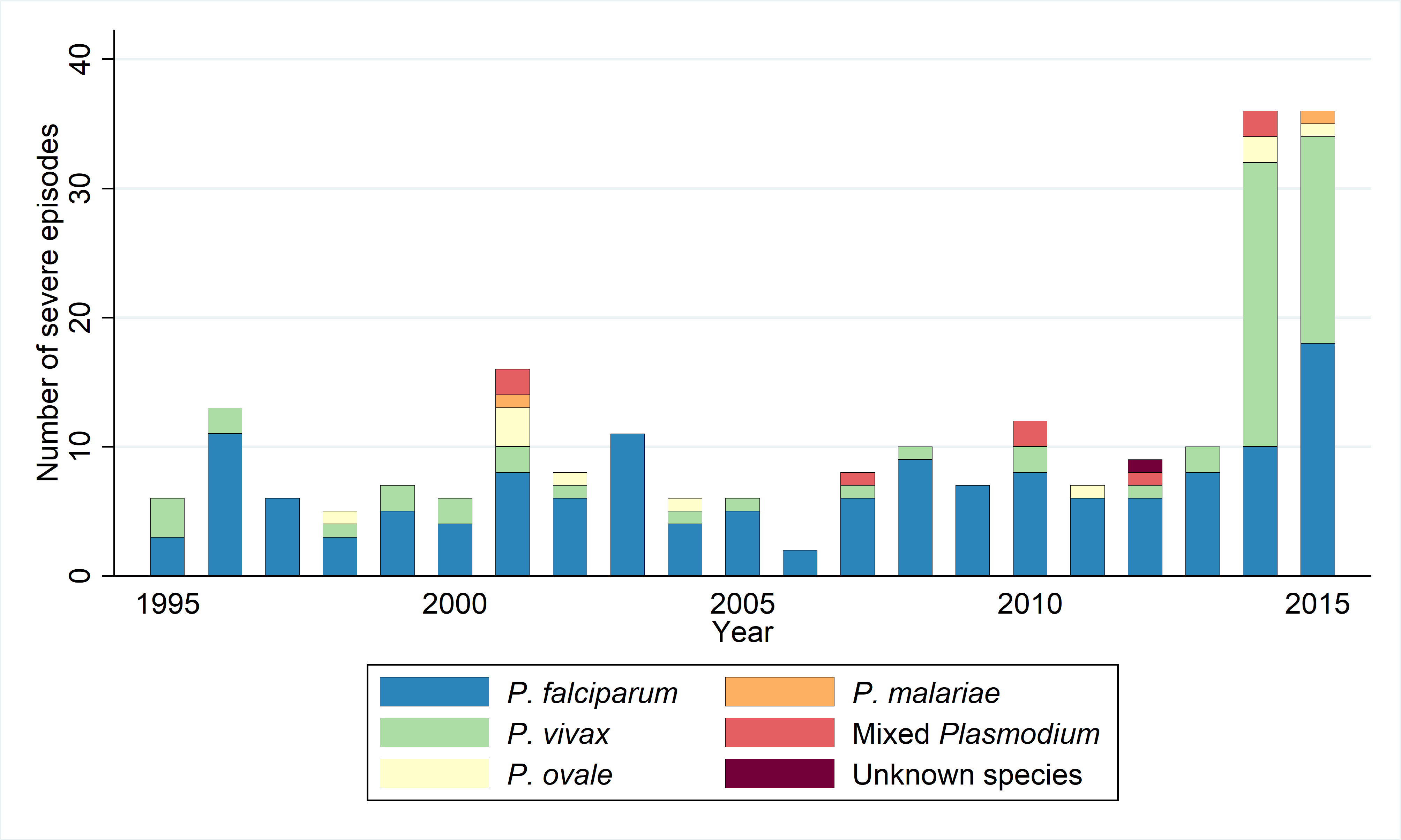

Supplement: jiz292_suppl_Supplementary_Figure_1c [file jiz292_suppl_supplementary_figure_1c.png]
