# Supplementary material for: Severity of Plasmodium falciparum and Non-falciparum Malaria in Travelers and Migrants: A Nationwide Observational Study Over 2 Decades in Sweden
Source: J Infect Dis. 2019 Jun 6;220(8):1335–45. doi: 10.1093/infdis/jiz292 (PMC6743839; doi:10.1093/infdis/jiz292)
Supplement: jiz292_suppl_supplementary_Table_1 [file jiz292_suppl_supplementary_table_1.docx]

**Supplementary table 1**

| Criteria for severe malaria according to WHO 2015 [2] |
| --- |
| Severe *falciparum* malaria  One or more of the following: |
| - Impaired consciousness – A Glasgow coma score <11 in adults or Blantyre coma score <3 in children |
| - Prostration – Generalized weakness so that the person is unable to sit, stand or walk without assistance |
| - Multiple convulsions – More than two episodes within 24 h |
| - Acidosis – A plasma base excess less than -3.3 mmol/L |
| - Hypoglycemia – Blood or plasma glucose <2.2 mmol/L |
| - Severe anemia – Hemoglobin concentration ≤50 g/dL in children <12 years of age and ≤70 in adults, with a parasite count of >10 000/µL |
| - Renal impairment – Plasma or serum creatinine >265 µmol/L |
| - Jaundice – Plasma or serum bilirubin >50µmol/L with a parasite count >100 000/µL |
| - Pulmonary edema – Radiologically confirmed or oxygen saturation <92% on room air with a respiratory rate >30/min |
| - Significant bleeding – Recurrent or prolonged bleeding from nose, gums, or venipuncture sites; hematemesis or melena. Splenic rupture. |
| - Shock^a^ – Systolic blood pressure <70 mm Hg in children or < 80 mm Hg in adults |
| - Hyperparasitemia – *P*. *falciparum* parasitemia > 10 % |
|  |
| Severe non-*falciparum* malaria^b^   - Same criteria as for *P*. *falciparum* but with no parasite density thresholds |

^a^ Modified from WHO 2015 [2], where compensated circulatory shock (capillary refill ≥ 3 s or temperature gradient on leg) is considered as a criterion for severe malaria.

^b^ WHO 2015 criteria for severe *P. vivax* was here extended to include all non-*falciparum* species.
