# Supplementary material for: Severity of Plasmodium falciparum and Non-falciparum Malaria in Travelers and Migrants: A Nationwide Observational Study Over 2 Decades in Sweden
Source: J Infect Dis. 2019 Jun 6;220(8):1335–45. doi: 10.1093/infdis/jiz292 (PMC6743839; doi:10.1093/infdis/jiz292)
Supplement: jiz292_suppl_Supplementary_Figure_Legend [file jiz292_suppl_supplementary_figure_legend.docx]

**Supplementary figure legend**

**Supplementary Figure 1.** Annual number of imported malaria cases of respective *Plasmodium* species between 1995-2015 in Sweden (**a**), proportion of travelers and migrants born in endemic or non-endemic countries (**b**), and number of severe malaria episodes in respective *Plasmodium* species (**c**).
